# Myocardial deformation and pro-arrhytmic indices in a group of first-episode patients with psychosis after one year of antipsychotic treatment

**DOI:** 10.1192/j.eurpsy.2025.830

**Published:** 2025-08-26

**Authors:** M. Plakoutsis, A. Bechlioulis, A. Rammos, S. Sioros, A. Karampas, G. Georgiou, D.-D. Koutsogianni, L. Mihalis, K. Naka, P. Petrikis

**Affiliations:** 1 Department of Psychiatry; 2 2nd Department of Cardiology, University of Ioannina, Ioannina, Greece

## Abstract

**Introduction:**

Heart Rate Variability assesses the autonomic system function. We have previously reported increased sympathetic activation (i.e. increased proarrhythmic risk) and asymptomatic left ventricular myocardial dysfunction in a group of drug-naïve, First Episode Patients with psychosis. We performed the same cardiological evaluation in a subgroup of these patients one year after the initiation of antipsychotic treatment.

**Objectives:**

To find out the impact of antipsychotic medications in cardiac function.

**Methods:**

Thirty three consecutive patients diagnosed with first psychotic episode were included in the current analysis. None of the patients had a previous history of cardiovascular diseases. All patients were assessed by 24-hour Holter ECG monitoring (including QT analysis and heart rate variability indices) and a thorough echocardiographic study (including myocardial strain analysis – global longitudinal strain GLS) at baseline (shortly after stabilization) and 1 year after treatment.

**Results:**

The mean age of our population was 29±7 years, 75% were males and their body surface area was 1.87±0.21 m^2^. At baseline 1) the mean value of QTc was normal in all subjects although the greatest QTc value measured was above 500msec in 35% of patients, 2) SDNNI<50 msec was observed in 50% of the patients, all patients had an abnormal HRV index, low RMSSD <20 msec was observed in 35% of patients, no patient had abnormally low PNN50 values. No patient had evidence of overt structural heart disease and the value of left ventricular ejection fraction was within normal range (57.5±5.1 %). GLS was decreased (i.e. < -16%) in 25% of patients. At follow-up maximum QTc value (517±52 vs 485±69, p=0.014) and PNN50 (14.1±12.1 vs 9.8±10.5, p=0.05) were increased while left atrial volume index (20.1±6.3 vs 23.3±6.4, p=0.031) and GLS (-18.6±2.5 vs -17.5±2.5, p=0.007) were decreased compared to baseline. No other significant changes were observed at follow-up. At follow-up, abnormal GLS, QTc max, SDNNI, HRV index, RMSSD and PNN50 values were observed in 16%, 60%, 38%, 96%, 15% and 0% respectively of included patients.

Normal values

SDNNI >100 (especially <50)

HRV INDEX >60

RMSSD 13-48 MS

Pnn50 -3 – 43%

**Conclusions:**

In patients with a first psychotic episode without a previous history of cardiovascular disease or risk factors, a significant proportion showed abnormal autonomous system function and subclinical myocardial dysfunction of the left ventricle. After 1 year of treatment, improvement was observed in left ventricular function and parasympathetic system activity with a concomitant increase QTc interval. Optimal management of psychotic episodes may lead to an amelioration of the risk of future myocardial impairment without affecting the risk for arrhythmias and sudden death.

**Disclosure of Interest:**

None Declared

